# Investigating client satisfaction with antiretroviral treatment services in South-South Nigeria

**DOI:** 10.1080/17290376.2019.1636709

**Published:** 2019-07-04

**Authors:** Omosivie Maduka

**Affiliations:** Department of Preventive and Social Medicine, University of Port Harcourt, Port Harcourt, Nigeria

**Keywords:** Satisfaction, qualitative, antiretroviral treatment

## Abstract

Client satisfaction is a key method of evaluating the quality of healthcare services. This research investigated client satisfaction with anti-retroviral treatment services in selected outpatient facilities in Rivers State. This study was a qualitative study carried out in four antiretroviral treatment (ART) facilities in Rivers State, Nigeria. Researchers conducted nine Key Informant Interviews (KIIs), 25 In-depth interviews (IDIs) and eight Focus Group Discussion (FGDs) among 73 Persons Living with HIVs (PLHIVS) consisting of 31 males and 42 females, using a topic guide. Interviews were recorded, transcribed and analysed using thematic content analysis. Majority of study participants interviewed reported being very satisfied with confidentiality, health worker attitude, interpersonal communication, counselling, and availability of drugs. The major sources of dissatisfaction included overcrowding, long waiting time and inadequate/expensive laboratory services. Suggestions proffered for improving the quality of care at the centres included increasing staff strength at the treatment centres, improving the quality and cost of laboratory services, and infrastructure upgrade. This study demonstrates the role health workers and facility processes play in satisfaction with services at HIV treatment centres. Health workers, programme officers, and managers in HIV prevention, care and treatment need to pay attention to these issues if they would be successful in improving the quality of care for PLHIVs.

## 1 Introduction

Highly active antiretroviral treatment (HAART) has since its discovery been effective in the management of HIV. It has changed the outlook of the disease from what was previously thought to be a death sentence to a chronic illness (Amico & Orrell, 2013). Government and its international partners have invested a lot of resources into making HAART available free of charge at antiretroviral treatment centres in the country. These treatment centres are manned by health care workers who are expected to provide a broad spectrum of care to persons living with HIV (PLHIVs). Some services offered include testing and counselling, adherence counselling, treatment initiation, drug refill, laboratory investigation and medical consultation.

The quality of care provided in these treatment centres has been shown to be associated with client outcomes. One such outcome, which is also a key method of evaluating the quality of health care services, is client satisfaction. It is defined as the degree of congruence between expectation and accomplishment (Heidegger, Saal, & Nuebling, 2006; Sitzia & Wood, 1997). It is thus a measure of how well the processes of care measure up to client's expectations (Smith, 2005). Satisfaction is a very important outcome of the quality of care. However, is not easy to measure since it is based on the subjective perceptions of the client. Client satisfaction can be elicited for several aspects of care following the Donabedian model of structure, process, and outcome as the various components for assessing quality of care in health institutions (Donabedian, 1966). Some dimensions of satisfaction with care include waiting time, cost of services, attitude of staff, ambience of the environment, information and education, and availability of staff (J. E. Ware, Snyder, Wright, & Davies, 1983).

Several researchers have linked quality of care at HIV treatment centres with retention in care, adherence to treatment and viral suppression (Hardon et al., 2007; Olowookere, Fatiregun, Ladipo, & Akenova, 2012; Roberts, 2002; N. C. Ware, Wyatt, & Tugenberg, 2006; Webb, Pesata, Bower, Gill, & Pallija, 2001). Client satisfaction with health care services is a valid concept with constructs that speak to the quality of healthcare services (J. E. Ware et al., 1983). Satisfaction with HIV treatment is an important consideration for the achievement of prevention and treatment goals for HIV control.

While a lot of research has been carried out on satisfaction of PLHIVs with their treatment centres, this research is concentrated in developed countries and is quantitative in approach. Furthermore, most of the qualitative studies identified focused on client perspectives alone without seeking out the views of healthcare staff at HIV treatment facilities. This research is, therefore, an attempt to bridge these gaps and add Rivers state-specific information to the existing body of knowledge on this topic. The findings from this research will prove useful to health care workers in HIV treatment facilities, facility heads, HIV control programme officers and health development practitioners as a whole.

The objective of this research was to investigate client satisfaction with specific aspects of antiretroviral treatment services, identify reasons for the satisfaction expressed by clients, and elicit suggestions for improving satisfaction.

## 2. Methods

### 2.1. Study area

Rivers State is one of the 36 states in Nigeria. It is located in the oil-rich region of the Niger Delta. The geography of the region is divided into upland and riverine with 23 Local Government Areas (districts); four of which can be said to be urban while the rest are mostly made up of rural settlements.

### 2.2. Study sites and sample selection

This study was carried out between August and November 2015 in four antiretroviral treatment facilities in Rivers State, Nigeria. Two study sites each were selected purposively from the clusters of government and NGO managed facilities to ensure representation of ART clients receiving care under the various service delivery models in the state. Facilities selected for the study include Health of the Sick (HOSH), Bori General Hospital (BGH), Niger Hospital (NH) and University of Port Harcourt Teaching Hospital (UPTH). Clients who were enrolled into the study were also sampled purposively to reflect a variety of client characteristics such as sex, literacy levels, socio-economic class, social support systems and adherence to treatment. This was done in order to include all possible perspectives.

Adult clients who gave informed consent and had had at least one visit to the treatment facility in the previous six months were considered eligible for the study. Clinic attendance records were used to identify clients who had defaulted from clinic visits and efforts made via telephone to recruit some of them for the study. This was done in an attempt to reflect the perspectives of this group regarding satisfaction with treatment services.

### 2.3. Study design and data collection

A descriptive study design was employed using qualitative methods consisting of focus group discussions (FGDs), key informant interviews (KIIs) and in-depth interviews (IDIs).

A minimum of two focus group discussions (one male and one female), six in-depth interviews and two key informant interviews were conducted per site. Each focus group had between seven to ten participants, and each session ran for 60–90 minutes. Each session had a moderator (the researcher or a trained assistant) and a note taker (research assistant). All focus groups used the same guide and addressed the same issues.

A minimum of six in-depth interviews were conducted per site; four with regular clinic attendees and two with clients who had defaulted in their clinic attendance in the preceding six months. This was done to be able to identify ideas and thoughts of persons actively utilising HAART services and those not optimally utilising services. A minimum of two key informant interviews were conducted with an executive of the HIV-AIDS support group and the health personnel in charge of service delivery at each facility.

All focus groups and interviews were scheduled outside of clinic appointments. A neutral venue (as chosen by the interviewees) outside of the facility was used for the focus group discussions. This was so that they could freely discuss any concerns or dissatisfaction they might have with facility staff. No financial remuneration was offered to study participants except a refund of transportation costs. Light refreshments were made available to participants during the focus group discussions. The researcher also obtained and distributed Information Education and Communication (IEC) leaflets on ‘positive living-life after HIV’ produced for PLHIVs by the Society for Family Health (SFH) after each focus group discussion.

The topic guide explored the perception of FGD participants, KII and IDI interviewees about their satisfaction with various aspects of care such as confidentiality, waiting time, attitude of staff, availability of drugs, the environment at the treatment facility and laboratory services. Other issues raised in the topic guide was the perceived reasons for the satisfaction or dissatisfaction with services at the treatment centre and recommendations for improvement.

The researcher developed a draft of the focus group discussion topic guide based on the study objectives and pre-tested it by conducting a focus group discussion with four persons living with HIV in the state. Their responses, opinions, and suggestions were used to modify the guide before use. The researcher also developed an interview guide for all key informant interviews and in-depth interviews. These guides were pre-tested with two interviews and subsequently modified. All topic guides were designed by the researcher based on the study objectives. Two research assistants who are junior residents in the Department of Community Medicine, University of Port Harcourt Teaching Hospital worked with the researcher to conduct the interviews and focus groups. These assistants had received HIV testing and counselling training and were also conversant with conducting focus group discussions. The researcher, however, conducted a one-day training for them on the objectives and methodology of this particular study. All discussions and interviews were recorded and transcribed in addition to the notes taken during the sessions.

The researcher conducted a thematic content analysis on the transcribed documents and notes for all interviews and discussions. This analysis focused on the responses to all interview and discussion questions across all respondents to identify themes and their variations/similarities across responses. The researcher identified and categorised emerging themes from recurring responses, and coded these themes. By doing this the researcher was able to identify patterns and relationships between these categories, and finally, interpret the data in a summary narrative.

### 2.4. Ethical considerations

The researcher obtained ethical approval from the ethics committee of the University of Port Harcourt Teaching Hospital, secured letters of support from the treatment centres used for the study and obtained verbal informed consent from all study participants after the study objectives and methods had been explained.

## 3. Results

The research team conducted nine KIIs, 25 IDIs, and eight FGDs among 73 PLHIVS (31 males and 42 females).

### 3.1. Satisfaction with specific aspects of care

During analysis of the focus group discussions, the key informant interviews and the in-depth interviews the following sub-themes were recurrent.

#### 3.1.1. Waiting time

Many clients and staff interviewed at the treatment facilities reported large crowds of clients at the treatment centres far more than staff numbers. The large crowds in their opinion resulted in long waiting hours before consultations or drug refills.

Many of them are not happy and because many of them will be eager to go home. We have more than 5000 clients in Bori here, and every day we serve more than 100 clients, so they outnumber the workers, some people they are not happy that they spend 3-5 hours in this place. (Support Group Leader HOSH during IDI)

On the issue of waiting time, it depends on the number of clients that visit the facility and the number of health workers available to serve them. Since most of our facilities are overcrowded, it means that clients have to be patient and wait until it gets to their turn. There is not much anyone can do about the current situation right now. (Support Group Executive during KII)

Waiting was particularly challenging at Bori GH, HOSH, and UPTH. However, clients and staff interviewed at Niger Hospital did not have this challenge.

*Unlike other ART centers, we don't have crowd in this place. People don*'t wait for long before they see the doctor or counselor and collect their drugs. The time we wait here to collect our drugs or to see the doctor is ok. (Client at Niger Hospital during FGD)

Clients interviewed at UPTH were full of commendations for the measures management had put in place to improve client flow and minimise waiting time.

*In this case, the procedure is perfect. When you drop your small card, you don't even know the person who is going to pick the card; some people are here calling names of the files that already out, others are there who have already collected the file, searching for it again. You don*'t even know when they will give it to you, no harassment, no stigmatization. (Client at UPTH during FGD)

#### 3.1.2. Drug availability

Clients and staff interviewed reported consistent supply of ART medication in the preceding three years. However, participants reported fluctuations in supply of medication during the immediate year of the study (2015). Most facilities claimed that they managed the challenge by reducing the quantity of pills given (from three months’ worth to one month's worth) so that more people could be covered in the short term.

*We didn*'t use to have problems with drugs until this year. Currently, we have been experiencing shortfall in the supply of our drugs so instead of giving our clients a three-months drugs regimen, we have adopted giving them drugs that will last for only one months. This strategy is to ensure that everyone has drugs especially ARTs pending when our drug supply improve. (Nurse, UPTH during KII)

*They always have drugs for us in this hospital. Every time I come here I always collect drugs. There has not been any time they didn*'t have drugs in this clinic. The government is really trying to make sure they have our drugs all the time; we thank God for them. (Client at UPTH during IDI)

For the past three years drugs have been very available, but we entered this year it has been gallop, it is no longer regular as at yesterday, there was no drugs. Last week our program supervisor had to run down to Uyo to go and borrow some drugs, so till now we don't have drugs especially the ARTs and the routine drugs, we don't have them. (Nurse Bori General Hospital during KII)

For this hospital, our drugs are always available especially the ART. We have never run short of it; we always have it. It is this period that we are having a little challenge but we still collect our drugs. (Client at HOSH during FGD)

#### 3.1.3. Confidentiality

Clients from all facilities interviewed were very happy about the level of confidentiality they enjoyed at all the treatment facilities. They shared reasons for their satisfaction with the level of confidentiality they enjoyed at the treatment centre and cited instances where confidentiality had been maintained to their satisfaction

The way I see it according to what they usually say, whatever you tell them remains a secret. Why I say it is secret is that you know this HIV matter, immediately you tell them, they ask us to make sure no one sees the container of the drugs we are taking and that the drugs must remain with you only. That's why I said they keep our secret. Anytime you come and want to see a doctor, they will make way for you to see the doctor. (Client at HOSH during FGD)

I can rate the confidentiality of services at these centers very high. We hardly hear reports of stigma from health workers on their clients. From my experience and the reports we get I can say that HIV treatment center in Rivers state ensure the confidentiality of their clients. Reported cases of health workers stigmatizing their clients are very rare these days. At most treatment center that I know the word HIV is rarely used when addressing clients publicly. (Support Group executive during KII)

*It is very confidential here. Because even when they are discussing with you, they don't allow someone else to be there, it's only you and the doctor. I don*'t think they discuss your issue with anyone else. Their services here are excellent. In fact, to me, I used to believe that this kind of service can be found in very developed countries. (Client at UPTH during IDI)

#### 3.1.4. Attitude of health workers

Most of the clients interviewed in the four treatment facilities expressed satisfaction with the attitude of staff. Their opinion was that most of the staff were courteous, friendly and kind and had good communication skills.

They talk to us very well, they don't shout at us, and they give us advice. They advise us to take our drugs regularly, taking care of myself by eating well, to avoid infections and other HIV related issues and information. (Client at General Hospital Bori during FGD)

I like it 100 times; I like them very well. The reason why I like them is that any time you come here, they will receive you very fine. They will attend to you and ensure that your needs are meet. They don't discriminate against anybody. (Client at HOSH during IDI)

Most of the health workers at HIV treatment center in Rivers have developed a good attitude towards PLHIVs. At the center where I get my drugs the staff, there are very friendly and willing to attend to the needs of their clients without asking for anything in return. So I can give them 70 percent regarding attitude towards PLHIVs. (Support Group Executive during KII)

#### 3.1.5. User fees

Clients interviewed had differing viewpoints about user fees and their satisfaction with the amounts charged for different services. There was consensus that most facilities collect a token of $0.55–$2.78 from each client during each clinic visit, baseline and follow-up investigations attracted varying user fees sometimes as high as N20,000, while drugs were given free of charge. Many felt that the costs for investigations should not be borne by the clients but defrayed in part or whole by government.

*Well most of our services here are provided free of charge, but we had to introduce the payment of* $0.55*. As I said earlier, we use this money to take care of some of the operational costs. We cover a large volume of clients here over five thousand, we require hands to help, and they receive allowances, we also have to provide basic office equipment and chairs for the clients. This* $0.55 *is just to help reduce the burden*. (Client at Niger Hospital during FGD)

In most of our hospitals especially those owned by government, you’re made to pay certain amount to get a card, and you also pay for any test you want to do. It is the same thing at treatment centers but the fees charged is not the same at all the facilities. Some of our clients sometimes find it hard to pay for these services especially the CD4 test so we think that government should do something to reduce the burden of our members some of whom are currently jobless. (Support Group Executive during KII)

The drugs they give us here is free of charge. Apart from the drugs, we pay for the CD4 test that we do here. (Client at UPTH during IDI)

#### 3.1.6. Information/counselling

Most persons agreed that health information, education, and communication were given priority at their treatment centres. Individual counselling was also being practised and was available either on the request of the client or initiated by the health worker. Issues commonly addressed during the group education and individual counselling sessions include drug adherence, safe sex practices, prevention and treatment of opportunistic infections, hygiene, and positive living. However, the support group executive interviewed complained about constraints to provision of adequate counselling services.

At most of the treatment centers where I have been to, the clinic starts with a talk which is normally on a HIV related issues like positive living, hygiene and diarrhea management … Clients who come early usually gain from these talks. Most of them also provide counseling services, but in some places, the counseling is not adequate because of the crowd that the counselors have to see. The counselors are also few in number and the environment may not be conducive for proper counseling. (Support Group Executive during KII)

*Sometime ago I didn't come for my drugs for about a month, they asked me the reason why I had not been coming for my drugs, after telling them the reasons, they warned me to stop doing that. So the counseling and information we receive here has helped us to understand HIV better and the need to* take our drugs regularly. (Client at UPTH during FGD)

### 3.2. Perceived reasons for client satisfaction /dissatisfaction with services

#### 3.2.1. Reasons for satisfaction

Some of the recurring reasons expressed by clients interviewed during the FGDs and the IDIs for their satisfaction with services at their treatment centres included the facilitation of support group activities by the centre, provision of health information and counselling and the attitude of the health workers.

*Yes our support group holds once a month and each time we gather, we don*'t feel like going because looking at members of the support group, we’re like symbols of the group, everybody looks fresh and better, so each time we stand up to talk people look at us first so am happy with everybody, we’re doing fine. (Client at HOSH during IDI)

*This is the first time in the history of this place. This is the first time I can really appreciate something like this in the history of medical service. It's been a wonderful service starting from the doctors, nurses and even the pharmacist that dispense drugs. It*'s very very wonderful, the way they will politely talk to you. … they are giving a kind of preferential treatment to people who come here. (Client at UPTH during IDI)

#### 3.2.2. Reasons for dissatisfaction

The major sources of dissatisfaction as identified during the discussions and interviews include overcrowding, long waiting time and inadequate/expensive laboratory services

Yes, I can say am satisfied with the services, at least 90% of them. The only area am not satisfied is the lab, the lab they are not doing fine, but other areas are ok. The only area my support group people usually complain is the lab. The CD4 machine is not working. Throughout this year we have not run the CD4 machine and my support group people are suffering. (Client at Bori GH during FGD)

*As you can see our crowd here; when there is a crowd, people have to wait for their turn to go in and see the doctor, it*'s natural because the doctor cannot see everybody at the same time. They have to see one person at a time, so we have crowd here, … it takes time, and some clients actually complain about the time they spend here, but we cannot do otherwise because of the crowd. That is how it is. (Client at HOSH during FGD)

### 3.3. Recommendations for improvement

The recurring recommendations for improving services at the treatment facilities centred around the need to provide more staff at the treatment centres and improve the quality of laboratory services. Other recommendations were to decrease cost of laboratory services to clients and upgrade the infrastructure at the treatment centres such as provision of rooms for individual counselling, and chairs for the waiting area.

There is the need to upgrade most of our HIV treatment centers and hospitals in general. Most of them lack adequate laboratory investigation equipment. Government should employ and deploy more health workers at these centers and ensure regular training for them to ensure high-quality service delivery. PLHIVs who have the required educational level and training should also be recruited by the different ministries, departments, and agencies of government. (Nurse Niger Hospital)

I think they should have counseling rooms where we can see counselors with different problems without seeing the doctors. (Client at UPTH during FGD)

They should employ more doctors, nurses, and counselors to work in this unit. (Client at UPTH during IDI)

One area where I think they really need to improve is the lab. Sometimes before you collect your test result, it takes time, and they may even say that they want to collect your blood sample again. This is not good. (Client at HOSH during FGD)

## 4. Discussion

Majority of the study participants interviewed across the four treatment centres reported being very satisfied with the attitude of the health workers and their skills at interpersonal communication and counselling. They were particularly satisfied with the availability of drugs, though some expressed concerns about impending drug shortages. There was also consensus about the satisfactory practice of confidentiality. Furthermore, some more reasons for their professed satisfaction with services at their treatment centres included the facilitation of support group activities by the centre, provision of health information and counselling and the attitude of the health workers.

On the other hand, the major sources of dissatisfaction as identified during the discussions and interviews include overcrowding, long waiting time and inadequate/expensive laboratory services. Suggestions proffered for improving the quality of care at HIV treatment centres in the state centred around the need to provide more staff at the treatment centres, improve the quality and decrease the cost of laboratory services, and upgrade of the infrastructure at the treatment centres. Some findings relating to satisfaction with waiting time, and the facility environment differed across treatment centres, while other findings relating to satisfaction with confidentiality and attitude of staff and dissatisfaction with laboratory services cut across all treatment facilities.

These findings agree with other qualitative research such as that by Hardon et al. in which participants across three countries reported being very satisfied with services at their HIV treatment centre (Hardon et al., 2007). Research by Watermeyer et al. who identified staff attitude, good communication skills, good organisation of services including short waiting as major aspects of satisfaction with treatment services (Watermeyer, 2012). In a study carried out by Boehme et al., a perception that staff at the treatment centre are caring, competent and informative led to feelings of satisfaction among study participants (Boehme et al., 2012). In contrast to the above studies, one qualitative researcher identified the quality of physician-patient relationships as the only factor that influenced satisfaction with care at HIV treatment centres (Roberts, 2002).

A researcher working among PLHIVs in South Africa, identified inconvenient clinic hours, long queues, waiting time and disrespect from staff as the major sources of dissatisfaction with care at HIV treatment centres. However, in this study, participants seemed willing to bear with long waiting times before receiving drugs or medical consultations. However, study participants from both research efforts agreed on poor health worker attitude as a key determinant of dissatisfaction (Bogart et al., 2013). Other studies also highlighted the role of attitude of health workers, user fees, poor organisation of treatment services, and long waiting times in stirring up feelings of dissatisfaction (Hardon et al., 2007; Orner et al., 2008).

These findings underscore what may be a relative acceptance of crowds at the treatment facilities and long waiting hours on one hand but an intolerance of poor staff attitude, high user fees and unavailability of drugs on the other hand. The implications of this is that as long as the treatment and investigations continue to remain free, drugs remain available, and staff remain informative, courteous and kind, PLHIVs may not mind the wait. This same relative acceptance was demonstrated in a similar study in Southwestern Nigeria (Olowookere et al., 2012). Acceptance does not, however, imply that HIV treatment clinics should not be better staffed, and better organised to be both effective and efficient. Participants identified the need to employ more doctors and nurses and better organise laboratory services as what is needed to improve satisfaction with care at their HIV treatment centres.

Findings from this research carried out in 2015 still find relevance in 2018. The reasons proffered for satisfaction and dissatisfaction with HIV treatment services by study participants will be most useful to HIV programme planners and implementers who desire to improve client satisfaction through continuous quality improvement of HIV care and treatment services.

### 4.1. Limitations of the study

A major limitation in a qualitative study design is the subjective nature of information acquired. Also, other factors extrinsic to the care in the HIV treatment centre may affect satisfaction with HIV treatment services, for instance, disclosure of status to family and friends and having a support structure and network. This study did not evaluate these possibilities. It did not also seek to directly identify any links to treatment outcomes such as retention in care and adherence. These are possible aspects to note for a follow-up study. A mixed methods approach in which quantitative measures are used to quantify satisfaction and identify areas of satisfaction/dissatisfaction while qualitative methods are used to dig deeper into the reasons and perceptions behind the views stated may provide a more holistic approach to understanding client satisfaction with HIV treatment services.

## 5. Conclusion

This study demonstrates the role factors such as cost of treatment, availability of drugs, attitude of health workers, access to laboratory services, confidentiality and waiting time, have on satisfaction with services at the HIV treatment centres. Health workers, programme officers, and managers in HIV prevention, care and treatment need to pay attention to these issues if they would be successful in improving the quality of care for persons living with HIV.
